# Efficacy of two ethanol-based skin antiseptics on the forehead at shorter application times

**DOI:** 10.1186/1471-2180-7-85

**Published:** 2007-09-24

**Authors:** Günter Kampf, Frank-Albert Pitten, Peter Heeg, Bärbel Christiansen

**Affiliations:** 1 Bode Chemie GmbH & Co. KG, Scientific Affairs, Melanchthonstrasse 27, 22525 Hamburg, Germany; 2 Institute for Hygiene and Environmental Medicine, Ernst Moritz Arndt University, Walther-Rathenau-Str. 49a, 17489 Greifswald, Germany; 3 Institute for Hospital Hygiene and Infection Control, Siemensstrasse 18, 35394 Giessen, Germany; 4 Institute for Medical Microbiology and Hygiene, University Hospital, Elfriede-Aulhorn-Strasse 6, 72076 Tübingen, Germany; 5 Institute for Hygiene, University Hospital Schleswig-Holstein, Campus Kiel, Brunswiker Strasse 4, 24105 Kiel, Germany

## Abstract

**Background:**

Recent research suggests that alcohol-based skin antiseptics exhibit their efficacy on the resident skin flora of the forehead in less than 10 minutes. That is why we have looked at the efficacy of two ethanol-based skin antiseptics applied for 10, 2.5 and 2 minutes on skin with a high density of sebaceous glands. Each experiment was performed in a reference-controlled cross-over design with at least 20 participants. Application of isopropanol (70%, v/v) for 10 minutes to the forehead served as the reference treatment. The clear (skin antiseptic A) and coloured preparations (skin antiseptic B) contain 85% ethanol (w/w). Pre-values and post-values (immediately after the application and after 30 min) were obtained by swabbing a marked area of 5 cm^2^ for about 10 s. Swabs were vortexed in tryptic soy broth containing valid neutralizing agents. After serial dilution aliquots were spread on tryptic soy agar. Colonies were counted after incubation of plates at 36°C for 48 h. The mean log_10_ reduction of bacteria was calculated. The Wilcoxon matched-pairs signed-ranks test was used for a comparison of treatments.

**Results:**

Skin antiseptic A applied for 10 min was significantly more effective than the reference treatment. When applied for 2.5 min (three experiments) it was significantly more effective than the reference treatment immediately after application (2.7 versus 2.2 log_10_ reduction; p < 0.001) and equally effective after 30 min (2.8 versus 2.6 log_10_ reduction; p = 0.053). Skin antiseptic B applied for 2.5 min (three experiments) was significantly more effective than the reference treatment both immediately after application (2.3 versus 1.9 log_10_ reduction; p < 0.001) and after 30 min (2.5 versus 2.1 log_10_ reduction; p = 0.002).

**Conclusion:**

The clear and coloured skin antiseptics applied for 2.5 min on the skin of the forehead fulfilled the efficacy requirements for skin antisepsis. The shorter application time on skin with a high density of sebaceous glands will allow to act more efficiently in clinical practice.

## Background

Surgical site infections are among the most common infectious complications of treatment in the hospital [1]. Up to 5.2% (USA) or 7.2% (Germany) of surgical patients in hospitals develop a surgical site infection during or after hospital stay depending on the type of operation [2-4]. The rate for surgical site infections after specific indicator operations, however, is lower [5]. Surgical site infections have a huge economic impact because patients remain on average 6.5 days longer in the hospital and are five times as often re-admitted to the hospital. They are 60% more likely to be treated on an intensive care unit. A surgical site infection costs approximately € 3.000 [6], depending largely on the type of underlying operation [7]. The crude odds ratio for hospital deaths associated with surgical site infections is 2.67 [8]. The resident skin flora has also been described to cause serious infectious complications [9,10]. In order to reduce the risk for surgical site infections a skin disinfection prior to the penetration of the skin is recommended among many other measures [11].

In central Europe, preparations based on ethanol or isopropanol are commonly used for skin antisepsis. There is currently no European norm available to determine the efficacy of preparations for skin antisepsis. That is why the efficacy of a preparation is commonly determined according to the test method of the German Society for Hygiene and Microbiology (DGHM) which was first described in 1991 [12]. It requires the comparison to a reference treatment in a cross-over design [13]. On skin with a high density of sebaceous glands the application time in the test method is 10 minutes for both the skin antiseptic and the reference alcohol. That is why in clinical practice the recommended application time for a skin antiseptic is usually 10 minutes on skin with a high density of sebaceous glands. The efficacy of alcohol-based skin antiseptics applied for shorter application times has first been studied in 2006 (Kramer; unpublished data). It was demonstrated with some alcohol-based skin antiseptics that a 3 min application is equally effective on the forehead to a 10 min application.

Based on these findings we investigated the efficacy of two new skin antiseptics based on 85% ethanol on the resident flora of the forehead with application times of 10, 2.5 and 2 min.

## Methods

### Design and preparation of subjects

Each experiment was performed in a reference-controlled cross-over design [13]. A minimum of 20 volunteers was recruited per experiment. Only participants with healthy skin on the forehead were selected (no injury, eczema or other inflammatory skin disease). Subjects were excluded when they took antibiotics or had used a disinfectant or antiseptic solution within the last three days before an experiment. The hair was clipped or tied around so that it would not touch the skin of the forehead during the investigation. The skin of the forehead was randomly divided into five areas of approximately five cm^2^. One area was chosen to determine the baseline bacterial density. Two areas were used for application of the reference alcohol (10 minutes), one for each of the different sampling times. Another two test areas were used for application of the skin antiseptic (2, 2.5 or 10 minutes), one for each of the different sampling times. A cotton swab was soaked with the skin antiseptic and swabbed over the marked test field. The procedure was repeated up to five times in order to keep the skin moist with the skin antiseptic for the entire application time. Ethical approval for studying the efficacy for skin antisepsis was obtained from the ethics committee of the University Hospital Kiel, Germany. The study was conducted in accordance with the ethical principles that have their origins in the current version of the Declaration of Helsinki (52^nd ^WMA General Assembly, Edinburgh, Scotland, October 2000). Informed consent was obtained from each participant.

### Products and application

The following preparations were used: propan-2-ol (70%, v/v) as the reference alcohol, a clear skin antiseptic based on 85% [w/w] ethanol (skin antiseptic A), and a coloured skin antiseptic based on 85% [w/w] ethanol (skin antiseptic B). The coloured skin antiseptic contains in addition the dyes E 104 and E 131, the thickener polyvinyl pyrrolidon. Both skin antiseptics were manufactured by Bode Chemie GmbH & Co. KG, Hamburg, Germany.

The clear skin antiseptic has been shown before with an application time of 3 to 5 min to have a superior efficacy on the bacterial flora of the forehead (Kramer; unpublished data). In out study it was applied to the skin of the forehead for 10 min and application times shorter than 3 min in order to identify the shortest application time with an efficacy equivalent to the reference procedure. The coloured skin antiseptic was only investigated with the application time in which the clear skin antiseptic was found to have an efficacy equivalent to the reference procedure in order to demonstrate that the addition of the dyes and the thickener does not impair the efficacy significantly. The reference alcohol was always applied for 10 min to the skin of the forehead according to the testing guideline [13].

### Determination of the pre-values and post-values

Sampling and cultivation were done according to the test method of the German Society for Hygiene and Microbiology [13]. Each sampling area was marked so that the standard size of 5 cm^2 ^was clearly visible. A cotton swab was soaked in tryptic soy broth (TSB). The sampling area was rigorously rubbed for about 10 s as described before [14]. Care was taken to ensure that the swab was only rubbed within the marked skin area. The swab was transferred into 5 ml TSB containing a combination of neutralizing agents for inactivation of residual biocidal activity [15]. In laboratory A the following neutralizing agents were used: 3% Tween 80, 0.3% lecithin and 0.1% cysteine. In laboratories B and C the following neutralizing agents were used: 3% Tween 80, 3% saponine, 0.1% histidine and 0.1% cysteine. Both combinations of neutralizing agents were found to be valid for neutralization of 85% ethanol (data not shown). The tube was vortexed for 30 s with a high frequency. A serial dilution was done in TSB. From appropriate dilution steps aliquots of 1 ml were spread on tryptic soy agar (TSA) in duplicate.

### Disinfection phase

Two marked skin areas on the forehead were treated with the reference alcohol, two other ones with one of the two skin antiseptics. After each type of treatment two samples were taken (post-values). The first sample was taken immediately after completion of the application (10, 2.5 or 2 min after beginning of the application). The second sample was taken 30 minutes after beginning of the application (20, 27.5 or 28 min after completion of application). Between each product application, a rest period of at least one week elapsed in order to allow the reconstitution of normal skin flora.

### Calculation of bacterial reduction

The plates were incubated for a total of 48 h at 36°C, and the colony-forming units (CFU) from plates were counted. For calculation purposes, plate count values ≤ 300 CFU were accepted. Plate count values of 0 were reset to 1, because the log_10 _of 0 is undefined, and the log_10 _of 1 = 0. The weighted mean of CFU was calculated taking into account the number of CFU per plate and the corresponding dilution step. The weighted mean was multiplied by the dilution factor in order to obtain the number of CFU per mL in the sampling liquid. All pre- and post-values were expressed as log_10 _values. For each sample from each volunteer, the logarithmic reduction factor (RF) was calculated as the difference between the log_10 _baseline value and the log_10 _post-values.

### Statistics

A product is considered effective for skin antisepsis if the mean RF at both sampling times is not significantly lower than the corresponding mean RF of the 10 min reference treatment (one sided Wilcoxon matched-pairs signed-ranks test; p > 0.1). Differences of all other means were investigated by the two sided Wilcoxon matched-pairs signed-ranks test [13]. A p-value < 0.05 was chosen to indicate a significant difference.

## Results

### Application time of 10 min

Two experiments were performed. The clear skin antiseptic A reduced the bacterial density on the forehead by 2.72 to 2.89 log_10_-steps (10 min after beginning of the application) and 2.39 to 3.45 log_10_-steps (30 min after beginning of the application). The skin antiseptic was either equally effective or more effective in comparison to the reference procedure (Table 1). Overall, application of the clear skin antiseptic A for 10 min yielded a significantly higher reduction of the resident bacterial flora in comparison to the 10 min reference procedure (p < 0.01; Wilcoxon matched-pairs signed-rank test).

**Table 1:** Efficacy of a clear skin antiseptic on the forehead applied for 10 min

Study facility and number of subjects	Sampling time after beginning of application	Mean pre-value of bacterial density	RF (mean and stdev) of reference treatment (10 min exposure)	RF (mean and stdev) of clear skin antiseptic A (10 min exposure)	p-value
A (n = 20)	10 min	3.88 ± 0.79	2.15 ± 0.78	2.72 ± 0.99	0.036
	30 min		1.94 ± 1.02	2.39 ± 0.97	0.012
B (n = 20)	10 min	3.81 ± 0.45	2.44 ± 0.81	2.89 ± 0.70	0.10
	30 min		2.97 ± 0.96	3.45 ± 0.61	0.044
All (n = 40)	10 min	n.a.	2.29 ± 0.80	2.81 ± 0.85	0.005
	30 min		2.46 ± 1.11	2.92 ± 0.96	0.004

### Application time of 2.5 min

Three experiments were performed with the clear skin antiseptic A (Table 2). In study facility A, the clear skin antiseptic A was significantly more effective at both sampling points (p < 0.001). In study facility B, the clear skin antiseptic A was significantly more effective (2.5 min after beginning of the application: mean RF of 3.17 versus 2.79; p = 0.028) and equally effective 30 min after beginning of the application (mean RF of 2.92 versus 3.02; p = 0.58). In study facility C, the clear skin antiseptic A was equally effective (2.5 min after beginning of the application: mean RF of 1.86 versus 1.77; 30 min after beginning of the application: mean RF of 2.39 versus 2.40); both differences were not significant. Overall, application of the clear skin antiseptic A lead to a mean log_10_-reduction of 2.74 (2.5 min after beginning of the application) which is significantly higher compared to the 10 min reference disinfection (mean log_10_-reduction of 2.19; p < 0.001). 30 min after beginning of the application the clear skin antiseptic A was equally effective to the reference treatment (2.77 versus 2.59; p = 0.053).

**Table 2:** Efficacy of a clear skin antiseptic on the forehead applied for 2.5 min

Study facility and number of subjects	Sampling time after beginning of application	Mean pre-value of bacterial density	RF (mean and stdev) of reference treatment (10 min exposure)	RF (mean and stdev) of clear skin antiseptic A (2.5 min exposure)	p-value
A (n = 20)	10 min/2.5 min	3.97 ± 0.47	2.08 ± 0.86	3.33 ± 0.38	< 0.001
	30 min		2.36 ± 0.63	3.05 ± 0.49	< 0.001
B (n = 20)	10 min/2.5 min	3.57 ± 0.48	2.79 ± 0.92	3.17 ± 0.67	0.028
	30 min		3.02 ± 0.69	2.92 ± 0.69	0.58
C (n = 23)	10 min/2.5 min	4.21 ± 0.71	1.77 ± 1.18	1.86 ± 1.40	0.51
	30 min		2.40 ± 1.29	2.39 ± 1.49	0.84
All (n = 63)	10 min	n.a.	2.19 ± 1.08	2.74 ± 1.16	< 0.001
	30 min		2.59 ± 0.97	2.77 ± 1.04	0.053

The coloured skin antiseptic B was studied in three experiments (Table 3). In study facility A application of the coloured skin antiseptic B was significantly more effective than the 10 min reference procedure both 2.5 min after beginning of the application (3.25 versus 2.50 log_10 _reduction; p < 0.001) and after 30 min (2.96 versus 2.59 log_10 _reduction; p = 0.006). In study facility B it was also significantly more effective both 2.5 min after beginning of the application (2.45 versus 1.94 log_10 _reduction; p = 0.007) and 30 min after beginning of the application (2.43 versus 1.86 log_10 _reduction; p = 0.01). In study facility C it was equally effective 2.5 min after beginning of the application (1.21 versus 1.26 log_10 _reduction; p = 0.60) and 30 min after beginning of the application (2.09 versus 1.93 log_10 _reduction; p = 0.83). Overall, application of the coloured skin antiseptic B for 2.5 min yielded a significantly higher reduction of the resident bacterial flora in comparison to the 10 min reference procedure (p < 0.05).

**Table 3:** Efficacy of a coloured skin antiseptic on the forehead applied for 2.5 min

Study facility and number of subjects	Sampling time after beginning of application	Mean pre-value of bacterial density	RF (mean and stdev) of reference treatment (10 min exposure)	RF (mean and stdev) of coloured skin antiseptic B (2.5 min exposure)	p-value
A (n = 20)	10 min/2.5 min	3.79 ± 0.54	2.50 ± 0.70	3.25 ± 0.50	< 0.001
	30 min		2.59 ± 0.69	2.96 ± 0.57	0.006
B (n = 20)	10 min/2.5 min	3.36 ± 0.81	1.94 ± 0.59	2.45 ± 0.81	0.007
	30 min		1.86 ± 0.74	2.43 ± 1.08	0.01
C (n = 20)	10 min/2.5 min	4.72 ± 0.60	1.26 ± 0.78	1.21 ± 0.64	0.60
	30 min		1.93 ± 1.18	2.09 ± 1.44	0.83
All (n = 60)	10 min/2.5 min	n.a.	1.90 ± 0.85	2.30 ± 1.07	< 0.001
	30 min		2.13 ± 0.94	2.49 ± 1.13	0.002

### Application time of 2 min

Two experiments were performed with the clear skin antiseptic A (Table 4). In study facility A, the clear skin antiseptic A was significantly more effective at both sampling points (p ≤ 0.001). In study facility B, the clear skin antiseptic A was equally effective 2 min after beginning of the application (mean RF of 2.08 versus 2.03; p = 0.55) and significantly less effective 30 min after beginning of the application (mean RF of 2.13 versus 2.53; p < 0.1). Overall, application of the clear skin antiseptic A lead to a mean log_10_-reduction of 2.57 (2 min after beginning of the application) which is significantly higher compared to the 10 min reference disinfection (mean log_10_-reduction of 2.05; p = 0.002). 30 min after beginning of the application the clear skin antiseptic A was equally effective to the reference treatment (2.59 versus 2.44 log_10 _reduction; p = 0.17).

**Table 4:** Efficacy of a clear skin antiseptic on the forehead applied for 2 min

Study facility and number of subjects	Sampling time after beginning of application	Mean pre-value of bacterial density	RF (mean and stdev) of reference treatment (10 min exposure)	RF (mean and stdev) of clear skin antiseptic A (2 min exposure)	p-value
A (n = 20)	10 min/2 min	3.97 ± 0.47	2.08 ± 0.86	3.05 ± 0.40	0.001
	30 min		2.36 ± 0.63	3.05 ± 0.49	< 0.001
B (n = 20)	10 min/2 min	3.37 ± 0.62	2.03 ± 1.30	2.08 ± 0.88	0.55
	30 min		2.53 ± 1.07	2.13 ± 1.00	< 0.1
All (n = 40)	10 min	n.a.	2.05 ± 1.08	2.57 ± 0.84	0.002
	30 min		2.44 ± 0.87	2.59 ± 0.91	0.17

## Discussion

For the first time we were able to demonstrate that the efficacy of an ethanol-based skin antiseptic is achieved on the forehead within 2.5 min and does not require a 10 min application time. This could be shown for a clear and a coloured skin antiseptic both based on 85% (w/w) ethanol. Extending the application time with any of the two skin antiseptics beyond 2.5 min has, based on our data, no additional effect on the bacterial density and can therefore be regarded as unnecessary. Shorter application times for skin antiseptics have been recommended as long as there is sound evidence that the efficacy is equal to the reference procedure and the skin antiseptic is approved as a medicinal product [16].

The results obtained with the 10 min reference disinfection on the skin of the forehead indicate some outcome variability among the ten experiments. The lowest mean log_10 _reduction obtained with the reference alcohol, measured as the immediate effect, was 1.26 ± 0.78, the highest mean was 2.79 ± 0.92. Similar results for reference alcohol have been described before [14,17,18]. A certain outcome variability has to be expected when the effect of antiseptics is measured on the resident bacterial skin flora without an artificial contamination. A similar test principle can be found in surgical hand disinfection when the efficacy of a hand rub is also measured against the resident hand flora. The variability of the outcome obtained with a surgical hand disinfection reference procedure is also relatively high [19]. When hands, however, are artificially contaminated (e.g. with *Escherichia coli*) the variability of the reference procedure efficacy is remarkably lower which can be explained by the standardized size of the high inoculum [20,21]. That is why it is essential to study the efficacy of antiseptics in a cross-over design in order to better come around the inevitable outcome variability of the reference procedure.

The bacterial biota in normal superficial skin are highly diverse [22]. Especially the skin on the forehead is known to have a stable and dense bacterial population which is rather difficult to reduce [23,24]. This is most likely explained by the high number of sebaceous glands excreting lipids [25,26]. Alcohol, however, has been described to penetrate into the deeper layers of the stratum corneum where it exhibits an effect which is similar to the one on the surface of the stratum corneum [27]. Other investigators have also studied the efficacy of skin antiseptics on the resident bacterial flora of the forehead and found a reduction of the resident skin flora in the same range [28-30]. The skin of the forehead is known to be an area with a higher density of resident skin bacteria compared to other areas of human skin [31]. Similar mean reductions with various types of skin antiseptics were found on abdominal skin [32]. It is known that alcohol does not completely eliminate the resident flora on the forehead [33].

Most skin antiseptics in central Europe are currently recommended with a 10 min application time on skin with a high density of sebaceous glands [34]. The reason for the 10 min application time is that they are usually tested for efficacy according to the method of the DGHM which only allows a 10 min application time [13]. To our knowledge this is one of the first investigations with skin antiseptics to identify a shorter but equally effective application time for specific formulations. Recent changes in surgical hand disinfection indicate that there is currently a trend to identify the application time which is truly necessary for an antiseptic preparation to fulfill the defined efficacy requirements [35,36]. This development in surgical hand disinfection has also been recognized and accepted by the relevant infection control societies in Germany [37,38] and is in principal considered to be an important step for evidence-based recommendations in infection control [39].

The clinical advantage is substantial if the application time on skin with a high density of sebaceous glands is reduced from 10 to 2.5 min without any loss of efficacy. But this will only be possible for preparations with sound evidence that a short application time yields the same efficacy as a long application time. A short application time will allow to perform an intervention with less delay not only in neurosurgery or plastic facial surgery but especially in skin antisepsis prior to lumbar punctures, epidural or peridural anaesthesia which may be particularly valuable in emergency procedures.

## Conclusion

The clear and coloured skin antiseptics which were tested in our study and which were applied for 2.5 min on the skin of the forehead fulfilled the efficacy requirements for skin antisepsis. The shorter application time on skin with a high density of sebaceous glands will allow to act more efficiently in clinical practice.

## Competing interests

The first author is paid employee of Bode Chemie GmbH & Co. KG, Hamburg, Germany.

## Authors' contributions

GK designed the study, all authors analysed the data, wrote the manuscript, read and approved the final manuscript.
